# Supplementary material for: Seeing or believing in hyperplexed spatial proteomics via antibodies: New and old biases for an image-based technology
Source: Biol Imaging. 2024 Oct 23;4:e10. doi: 10.1017/S2633903X24000138 (PMC11503829; doi:10.1017/S2633903X24000138)
Supplement: Bolognesi et al. supplementary material 1 — Bolognesi et al. supplementary material [file S2633903X24000138sup001.docx]

**SUPPLEMENTARY DATA**

Title:

Seeing or believing in hyperplexed spatial proteomics via antibodies. New and old biases for an image-based technology.

Maddalena M Bolognesi, Lorenzo Dall’Olio, Amy Maerten, Simone Borghesi, Gastone Castellani, Giorgio Cattoretti

Gray tones discrimination test

The website <https://time.com/4663496/can-you-actually-see-50-different-shades-of-grey/> provides an evaluation of the discriminative power of the human eye for shades of gray.

A selection of eight new images are presented each accession, containing nine randomly assorted squares, over a background tone. Overall, gray tones ranging from 32 to 228 pixels are shown at any accession, the background tone ranges from 44 to 220 pixels, each 9-square screen displays gray levels, distant 47.9 ±15.2 pixels from the background (88-16), in 4 pixels increments/decrements from the background. These data derive from the analysis of five different tests.

A perfect score (all squares different from background selected and none of the ones identical to the background) is 50. Given these details, a score of 50 (obtained by none of the participants) requires the human eye to discriminate 2-5 background-toned squares among nine for 8 times in a row, whose gray tones are 4 pixels apart. This equals to a discrimination of 256/4 = 64 tones, which may be the top discriminative power of the human eye.

The technical characteristics of the computer screens used were not standardized.

Bit depth reduction discrimination test

Three micron sections were obtained from anonymous formalin-fixed, paraffin embedded surgical specimens, placed on glass slides and routinely processed for Hematoxylin and Eosin or for immunohistochemical markers in dedicated autostainers.

The Hamamatsu NanoZoomer S60 scanner (RRID:SCR_023762) (Hamamatsu Photonics K.K., Hamamatsu City, Japan) is equipped with a Nikon 20× or 40x/0.75 PlanSApo objective and a linear ORCA-Flash 4.0 digital CMOS camera (Hamamatsu Photonics K.K.).

Slides were scanned at 20x or 40x at a resolution of 0.45 and 0.25 µm/pixels respectively. Selected cases were acquired as 5-stacks zeta-stack and merged in a single-layer image via the Hamamatsu toolkit.

The whole slide images (WSI) were saved as .ndpi files at a resolution of 300 dpi.

Selected areas were opened with the NDP View 2.0 software (Hamamatsu Photonics) at the optical maximal magnification and saved at the original resolution (300 dpi) as .jpg images.

Still life images (as controls) were obtained with a Panasonic DMC-TZ100 camera equipped with a 20.1 megapixel MOS sensor. Images were saved as 16 bit RW2 files.

A bar with a continuous grayscale gradient was created in Microsoft Powerpoint (RRID:SCR_023631) and exported as a 2550 x 556 pixel image, 225 dpi.

Images were imported in Adobe Photoshop 25.0.0 (RRID:SCR_014199) as 8 bit per channel (RGB; 24 bits) and downsized to 7, 6, 5 and 4 bits via the Adjustments > Posterize command, then saved in the new format with the original size and resolution. The 7-bit image was not used for the test, except for the grayscale gradients bars.

Note that the pixel resolution did not change during this process.

An example of the bit depth degradation (or posterization) is shown in Fig. 2.

Bit channel depth will be defined throughout this manuscript referred to a single channel, i.e. 8 bit for maximal resolution (equals 24 bits in RGB channels).

Composite images and labeling were obtained with Adobe Illustrator (RRID:SCR_010279).

Full size four-images composites for scoring were uploaded into NDPserve (Hamamatsu Photonics) and a link provided for image visual analysis. The NDPserve image can be zoomed 2x on screen.

A low-power overview of the image panels is shown in Fig. S3.

Each observer was requested to assign the correct bit depth to each of the four images in each panel, via a pull-down fixed menu. One H&E and one IHC image was scored twice, adding a request to identify similar images among the 4-image panel.

For the continuous gray shaded bars, the observer would identify the bars showing laddering.

All pathologists scored all the images.

A fillable PDF scoring sheet (Adobe Acrobat Pro RRID:SCR_023361) with links to the full size images and pull-down scoring menus was provided to each participant. The PDF sheet contains a scoring result summary pages which can be exported to a spreadsheet for analysis.

The type of monitor used and resolution was recorded (see Supplementary material), but not used in the analysis.

The original images and the PDF test are available at Bicocca Open Archive for Research.

All the tests were performed on fully anonymous human specimens, in compliance with the Helsinki declaration for international guidelines for research. Steenbras (Lithognathus mormyrus) was purchased for human consumption at a local market, thus exempt from IRB. Flowers were a gift from a close relative. Non-pathology images were provided as familiar visual references for posterization.

Public CODEX data.

The image files and the .csv file for a human LN immunostained with the CODEX platform (now PhenoCycler, Akoya Biosciences, Delaware, USA) are accessed at the HubMap website (https://portal.hubmapconsortium.org/), and for the sample HBM754.WKLP.262 (doi:10.35079/HBM754.WKLP.262) at ​​<https://portal.hubmapconsortium.org/browse/dataset/c95d9373d698faf60a66ffdc27499fe1> , selecting, in sequence: drv_CX_20-008_lymphnode_n10_reg001 > processed_2020-12-2320-008LNn10r001 > the folder “stitched” > the folder “reg001” and then downloading the images as 16-bit .tiff (accessed last June 11, 2023).

Tyramide amplification (TSA)

Semi-serial sections from sentinel lymph nodes were selected for sensitivity comparison experiments.

Dewaxed, antigen retrieved FFPE sections were incubated for 10 min. with a H_2_O_2_ - NaN_3_ mixture ^(1)^ in order to block endogenous peroxidases. Afterward, the sections were washed in TBS-Ts and incubated overnight at RT with two primary antibodies, mismatched for species. The next day, the sections were washed twice for 15 min. In TBS-Ts without NaN_3_.

A blocking horse serum (Vector, Burlingame, CA) was applied for 10 min., rinsed once, and a secondary antibody HRP-conjugated polymer (Vector) was applied for 30 min.

After two 15 min. rinses in azide-free TBS-Ts, a freshly made TSA solution was applied for 20 min.. The TSA solution was made of 1 ml of Tris buffer pH 7.4, 10 µl of 100x H2O2 solution (made of 50 µl H2O2 3% in 1 ml distilled water) and 10 µl of the 100x Tyramide stock solution.

The Tyramide stock solution was made by dissolving the lyophilized product in DMSO as specified by the vendor (see Pub. No. MAN0015834, Invitrogen) and kept at -20 C°.

The TSA reaction was stopped by several rinses in TBS-Ts with NaN_3_, then the second primary antibody was counterstained for 30 min. with an appropriate secondary antibody. The staining cycle was repeated twice ^(2)^. After a final 30 min. wash, the section was counterstained with DAPI, mounted and scanned.

Fluorescence acquisition

The Hamamatsu NanoZoomer S60 scanner (Hamamatsu Photonics K.K., Hamamatsu City, Japan) is equipped with a Nikon 20×/0.75 PlanSApo objective, a Fluorescence Imaging Module equipped with a L11600 mercury lamp (Hamamatsu Photonics K.K.), a linear ORCA-Flash 4.0 digital CMOS camera (Hamamatsu Photonics K.K.) and two six-position filter wheels, one for excitation, the other for emission filters, and a three-cube turret. The filter setup is composed of a set of excitation filters, housed in one wheel: 387/11 (DAPI), 438/24 (BV480), 480/17 (FITC), 556/20 (TRITC), and 650/13 (Cy5); one set of emission filters: 435/40 (DAPI), 483/82 (BV480), 520/28 (FITC), 617/73 (TRITC), and 694/44 (Cy5). Dichroic filters are housed in three OM cubes: a triband FF403/497/574-Di01 (DAPI/FITC/TRITC) and two single pass FF458-Di02 (BV480) and FF655-Di01 (Cy5). All filters are from Semrock, Lake Forest, Ill, USA.

AlexaFluor 657 Tyramide was acquired for 96 milliseconds. Double indirect IF acquisition time ranged from 192 to 320 ms for Cy5 and 112 to 192 ms for FITC.

Image manipulation for viewing

Raw .tiff uncompressed files were produced by a Fiji plugin (Bio-format importer) from .ndpi grayscale fluorescence images.

Two types of image manipulations were used: log transformation and Brightness & Contrast.

For log transformation, the math>log command was applied to the dark background IF images, which were then inverted.

The Brightness & Contrast command was set to “auto” to inverted IF images.

Although both methods produce equivalent results, we found the log transformation the best one for images where the positive signal is most minuscule. For composite color images, both the log transformation and the Brightness & Contrast commands were used and adjusted visually.

The “Analyze” command “3D surface plot” was applied to modified IF images and the “Min” and “Max” cursors adjusted manually.

IHC/IF quantification

Image thresholding was used for IHC or IF quantification. The default setting for the thresholding algorithms in ImageJ/Fiji (<https://imagej.net/plugins/auto-threshold>) was used.

IHC immunostains of cytokeratin-positive FRC and of IF stains were quantified as single cells with QuPath (RRID:SCR_018257); Hematoxylin OD density was adjusted between 0.1 and 0.01, the nuclear outline was expanded by 0.1 µm, cytoplasmic DAB mean OD density was set at 0.1. Additional information can be found in the Supplementary Tables.

BRAQUE pipeline

BRAQUE is a python pipeline for automated cluster enhancing, identification, and characterization. Its code and documentation (comprehensive of usage instructions) can be found at https://github.com/LorenzoDallOlio/BRAQUE. Such pipeline can either be run on a tidy dataset in .csv format or its functionalities can be cherry-picked to be inserted into different pipelines. The HubMap dataset .csv was loaded and run after minor adjustments.

Phenograph pipeline.

The HubMap dataset .csv was analyzed with a Phenograph pipeline as published ^(3)^.

Intensity data for each biomarker and for each cell in the cluster are shown as hierarchical clustering plot (see ^(3)^); the custom R script can be downloaded at <https://board.unimib.it/datasets/46r9hcvpbd/1>. Note that mean intensity rendering as an heatmap may be deceiving ^(4)^.

SUPPLEMENTARY DATA AVAILABILITY

Data are deposited in Bicocca Open Archive Research Data (BOARD)

Bolognesi, Maddalena; Dall'Olio, Lorenzo; Maerten, Amy; Borghesi, Simone; Castellani, Gastone; Cattoretti, Giorgio (2023), “Seeing or believing in hyperplexed spatial proteomics via antibodies.”, Bicocca Open Archive Research Data, V1, doi: 10.17632/kmxz7fgydx

<https://data.mendeley.com/datasets/kmxz7fgydx/1>

SUPPLEMENTARY TABLES LEGENDS

One single Excel file contains the following tabs:

Primary and secondary Abs: Primary and secondary antibody specifications

Table-allCellsClassified: Classification of all BRAQUE clusters in the HubMap lymph node

Table-stromalCells: Classification of all BRAQUE stromal cell clusters in the HubMap lymph node

QuPathMeasurements-IHC_IF: Measurements and settings for QuPath

50shades-Survey: Observer's scores for the "50 shades of gray" website

PathologistsScoring: Scoring sheets and biometric info for all pathologists scoring bit-degraded images

BitDepth-chahnnelsEquivalence: Chart with equivalence calculations between bits, channels and colors.

VisionLimitsReferences: References to limitation of human visions, else than published papers.

SUPPLEMENTARY FIGURE LEGENDS


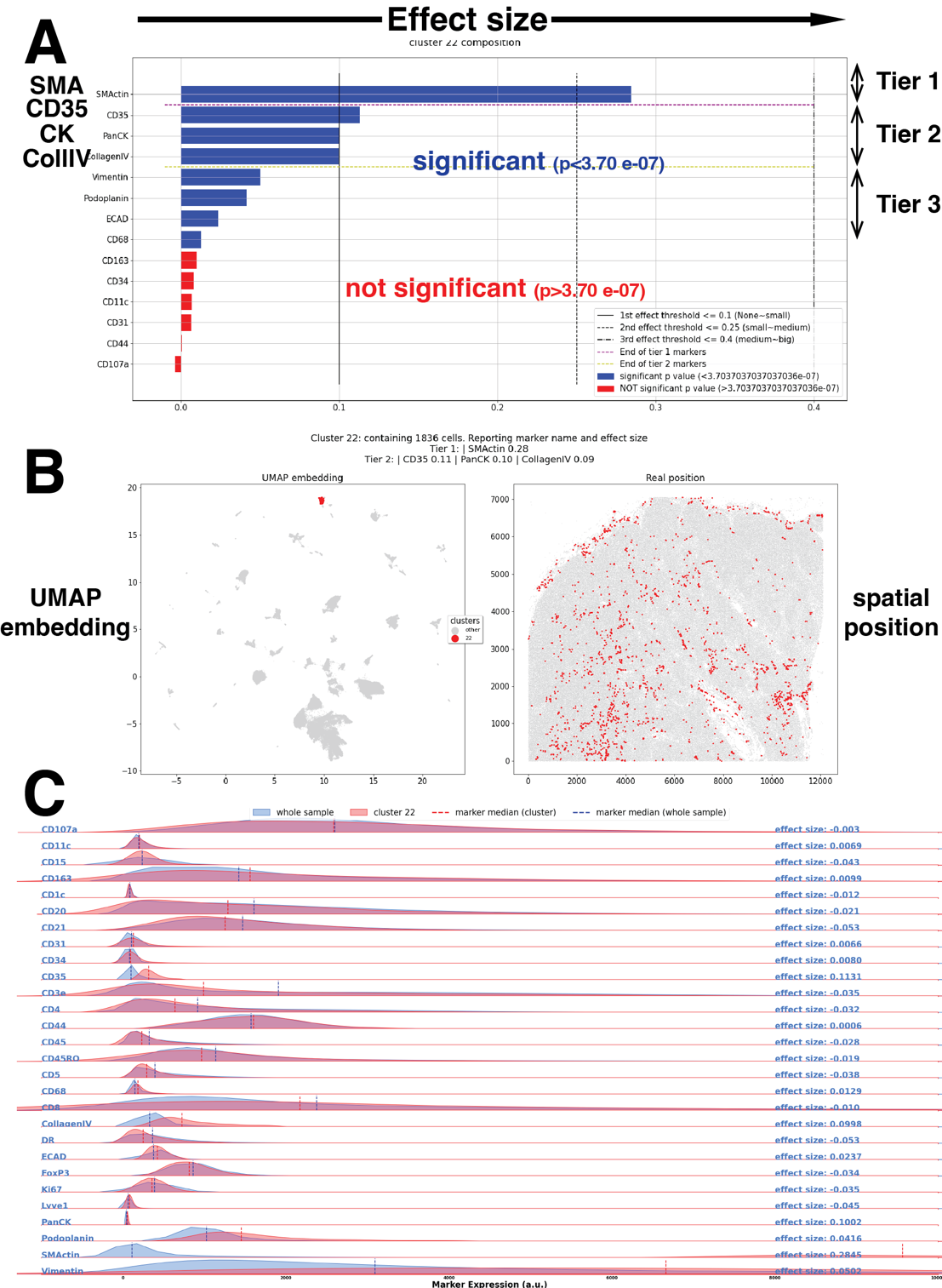


Fig. S1

BRAQUE display of an example of a FRC cluster (cluster #22).

**A** The markers defining cluster 22 are ranked according to the robust effect size metric 𝑑_𝑠𝑖𝑔𝑛𝑒𝑑_, and colored according to their Welch t-test p-value (blue: significant; red: non-significant). Note pan CK positioned in the Tier 2 position (“possibly” defining). **B** the position of Cluster 22 in the UMAP graph is shown on the left; the cells composing this cluster are superimposed as red dots on an image of the lymph node in grey, on the right. **C** the distribution of 26 markers in cluster 22 (red) is compared with the distribution on the whole LN (blue) in real values.


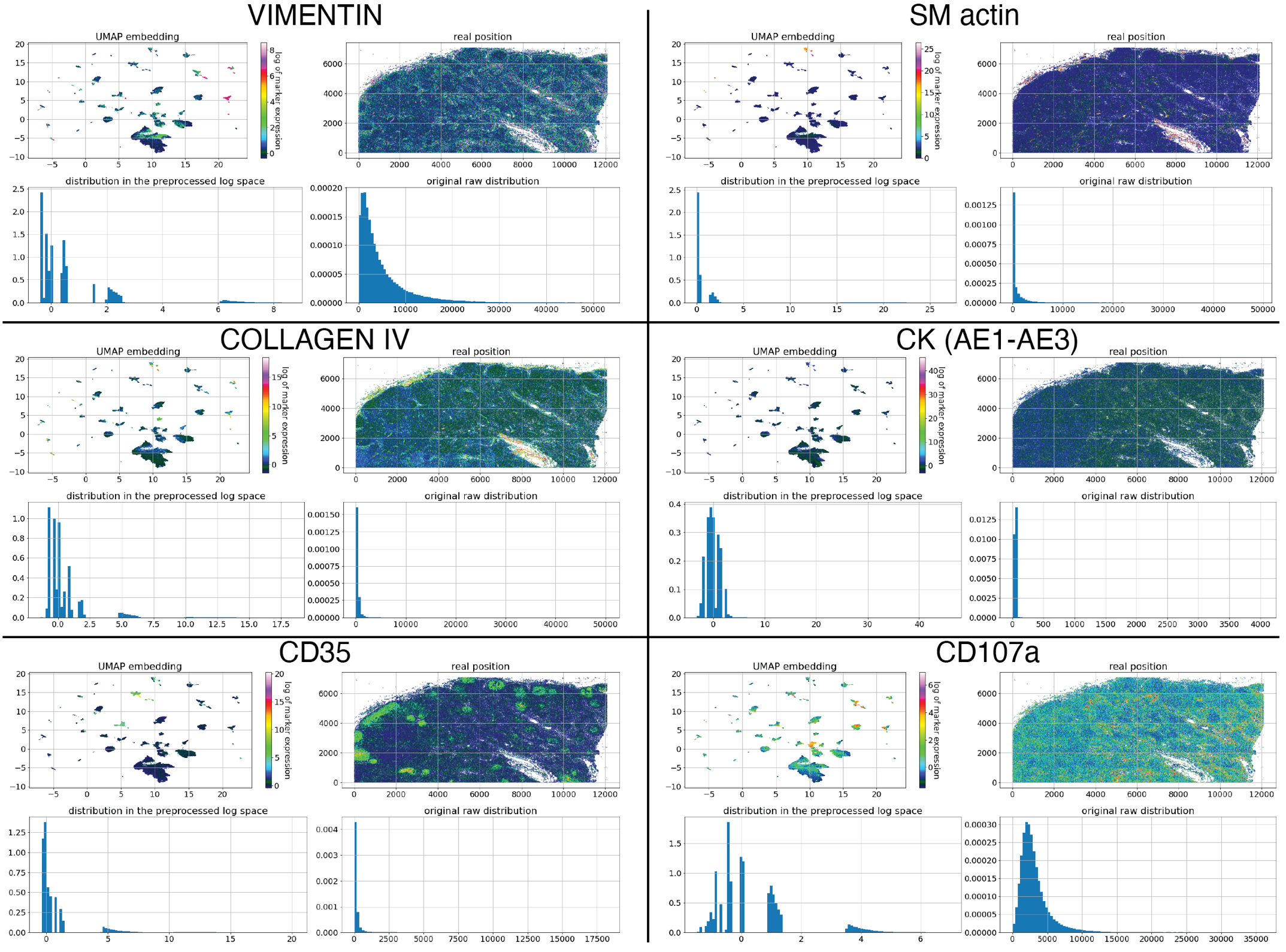


Fig. S2

Expression plots and distribution for FRC markers

BRAQUE display of six markers relevant for FRC classification. For each marker, in clockwise order, is presented: the marker log expression according to a color palette in the UMAP plot; the pseudocolor tissue distribution; the original raw intensity distribution; the distribution in the preprocessed log space after gaussian mix distribution and shrinking. Note the continuous data nature in the original raw distribution and the discretization in the preprocessed log space.


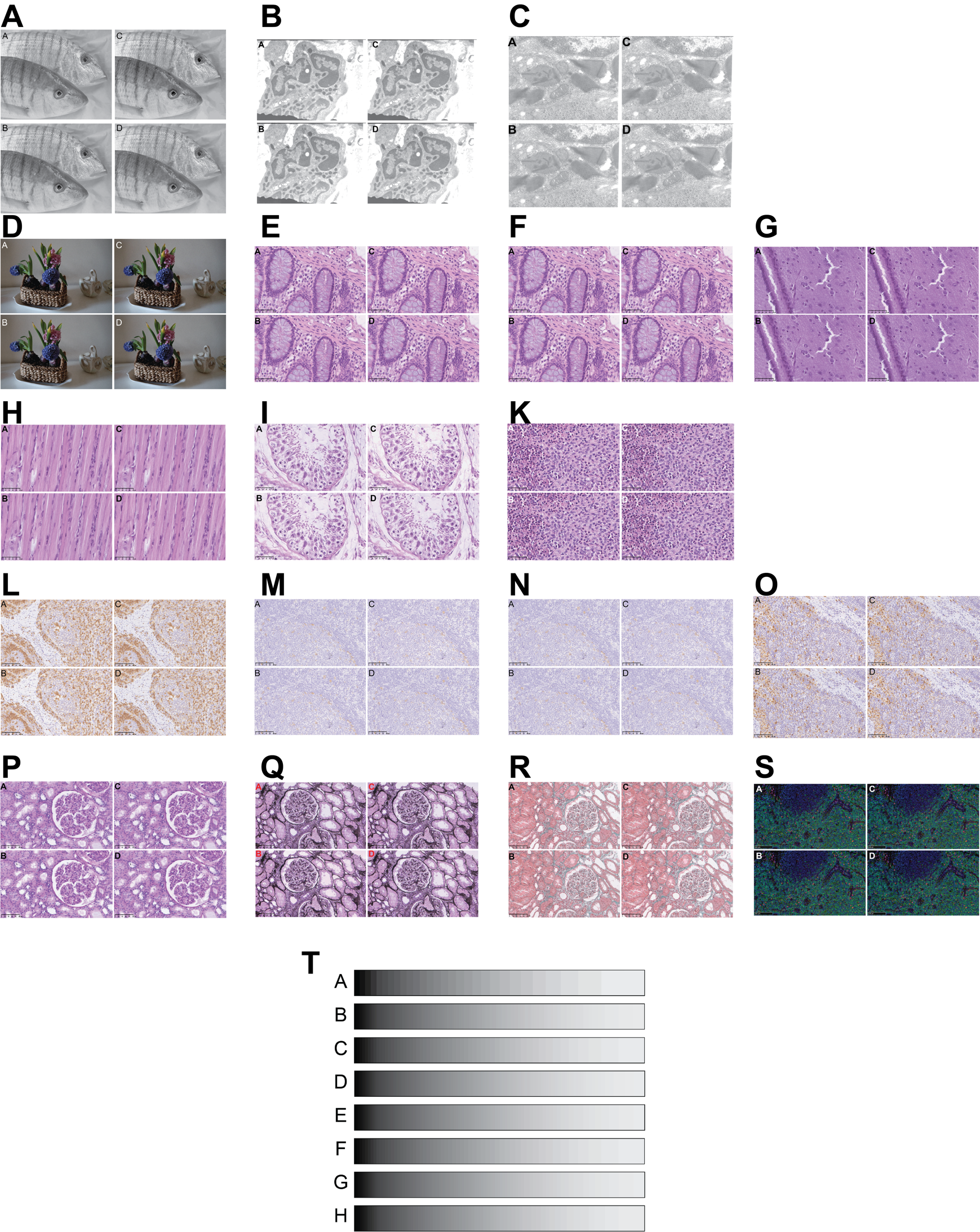


Fig. S3

Overview of the image panels tested.

Each subpanel consists of four images of different bit depth, the order scrambled for each set, to be scored online full-size. A: steenbras; B: electron microscopy image, low power; C:electron microscopy image, high power; D: flower pot, hyacinths; E, F: normal human colon; G: brain; H: muscle; I: testis; K: Langerhans cell histiocytosis (LCH); L: CD3 IHC on tonsil; M, N: CD30 IHC; O: CD68; P: PAS stain, kidney; Q: Jones stain, kidney; R: trichrome stain, kidney; S: triple immunofluorescence for CD4 (FITC), FOXP3 (TRITC) and DAPI, tonsil; T: monochrome bands, degraded with controls, scrambled.


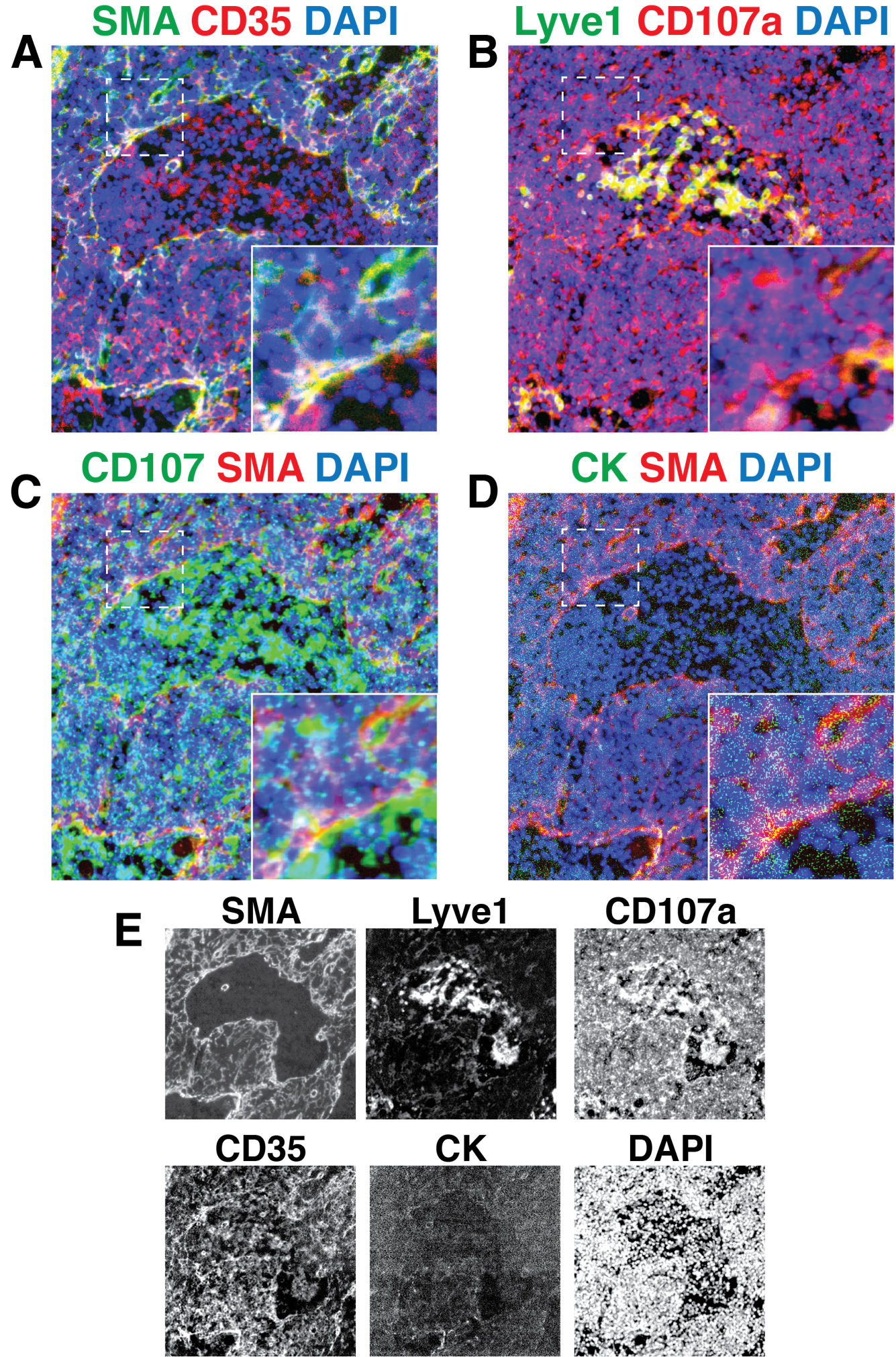


Fig. S4

Composite images for coexpression of CD35 or CD107a on FRC and Lyve1 endothelial cells.

A detail of the .tiff images for SMA, Lyve1, CD107a, CD35, CK and DAPI is superimposed in color composite. A detail highlighted by the dotted square is in the lower right corner of each image. **A**: SMA and CD35 are coexpressed (yellow) in FRC. **B**: Lyve1 and CD107a are coexpressed (yellow) in endothelial cells and in sinus macrophages. **C**; CD107a and SMA are juxtaposed, but do not colocalize, retaining the green or red color. **D**: CK staining is diffuse and dust-like over the field, occasionally co-localizing with SMA+ cells. **E**: greyscale single stain images of the composite components. All images have been adjusted with Fiji for brightness and contrast in order to be visible to the human eye. Scale bar: 100 µm


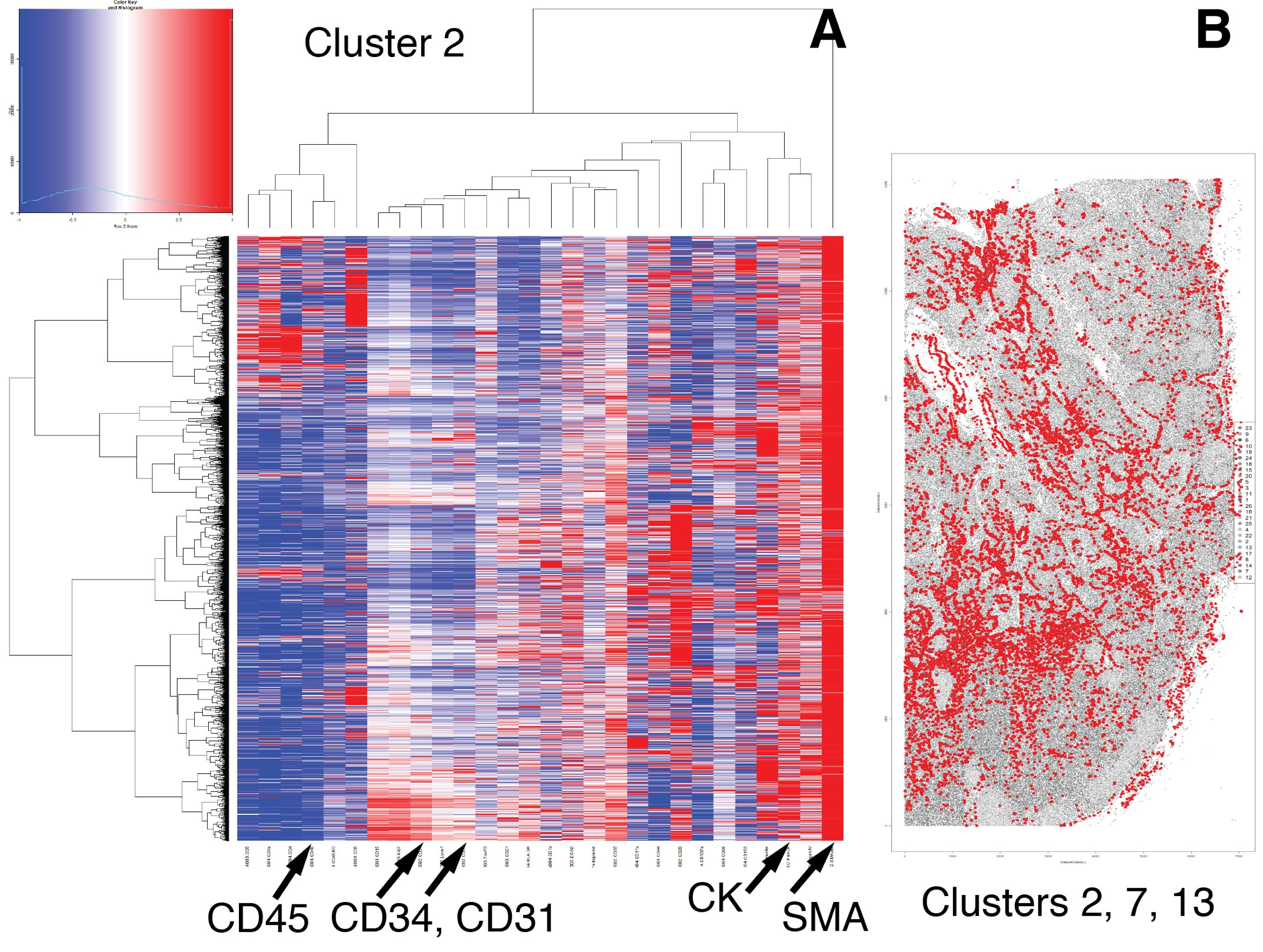


Fig. S5

Representative CK+ FRC cluster and tissue distribution by Phenograph.

**A**- a representative CK+ Phenograph cluster (cluster 2) contains SMA+ and CK+ cells, which are characterized by negative CD31, CD34 and CD45 expression. Rows are cells, columns are markers, ordered by hierarchical clustering. **B**- CK+ FRC clusters (2, 7 and 13) are plotted in red onto a gray image of the LN.


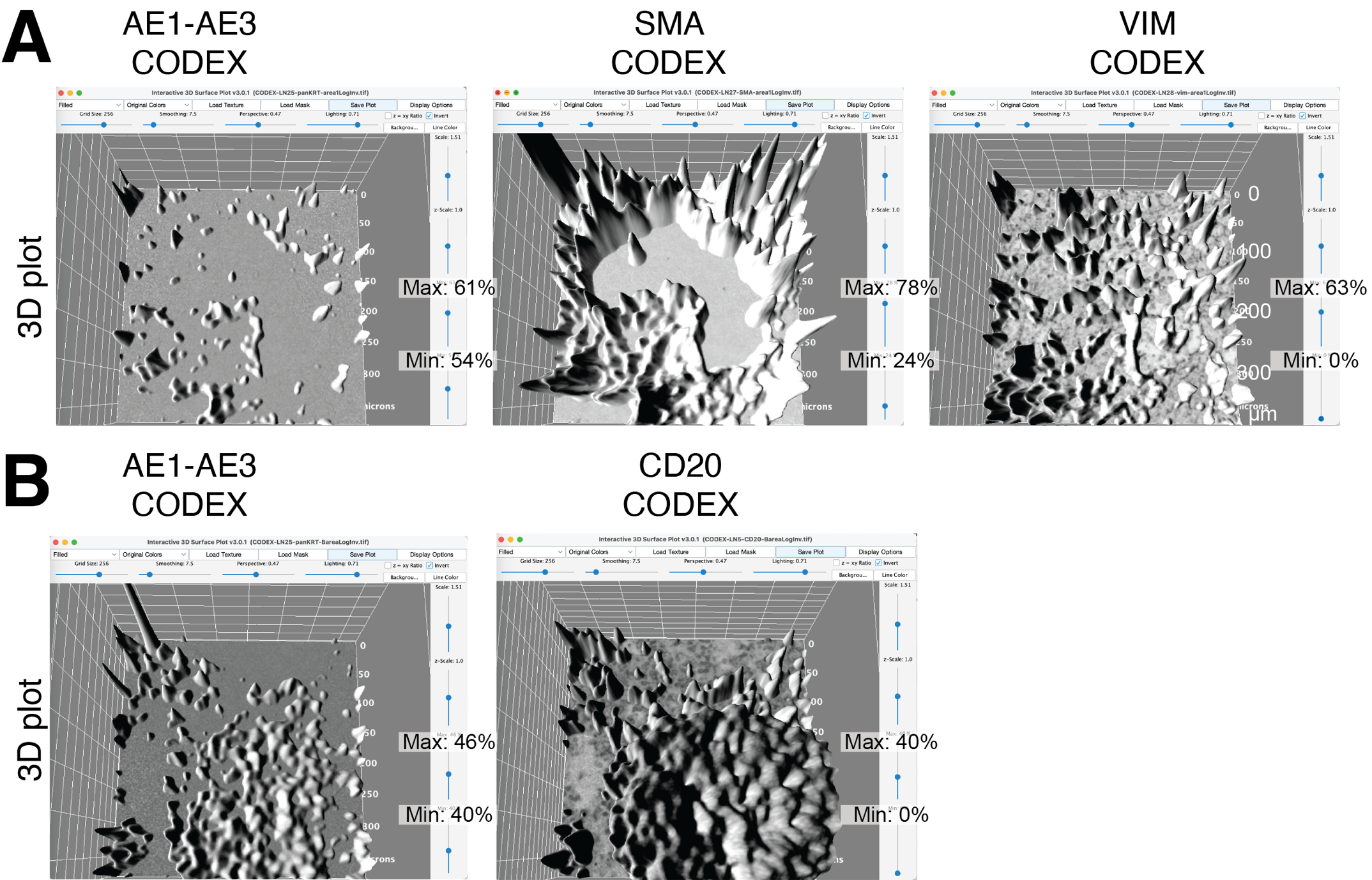


Fig. S6

The settings for the 3D plots of Figure 6 are listed for each image. Only setting variating in each image are magnified. Invariant settings on the x axis are: Grid Size: 256; Smoothing: 7.5; Perspective: 0.47; Lightning: 0.71; On the y axis: Scale: 1.51; z-scale: 1.0. The grid white numbers are microns. Note the individual manual adjustments in Min. and Max. values for each biomarker.


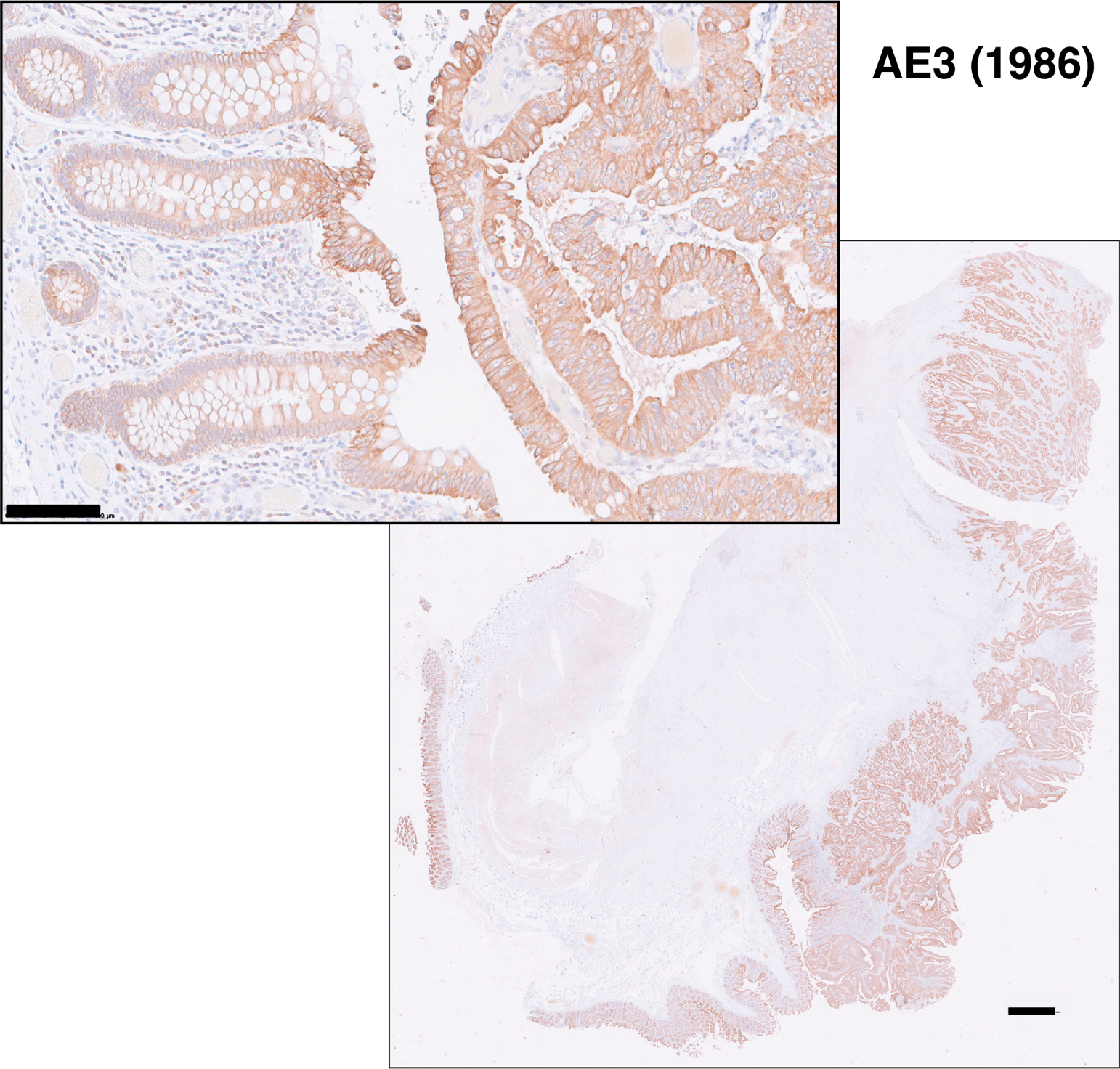


Fig S7.

Indirect immunoperoxidase staining (DAB) of a colon cancer specimen with the AE3 antibody obtained in 1986. DAB color is brown, hematoxylin nuclear blue counterstain. Scale bar for the low-power image: 1 mm; for the high magnification inset: 100 µm.


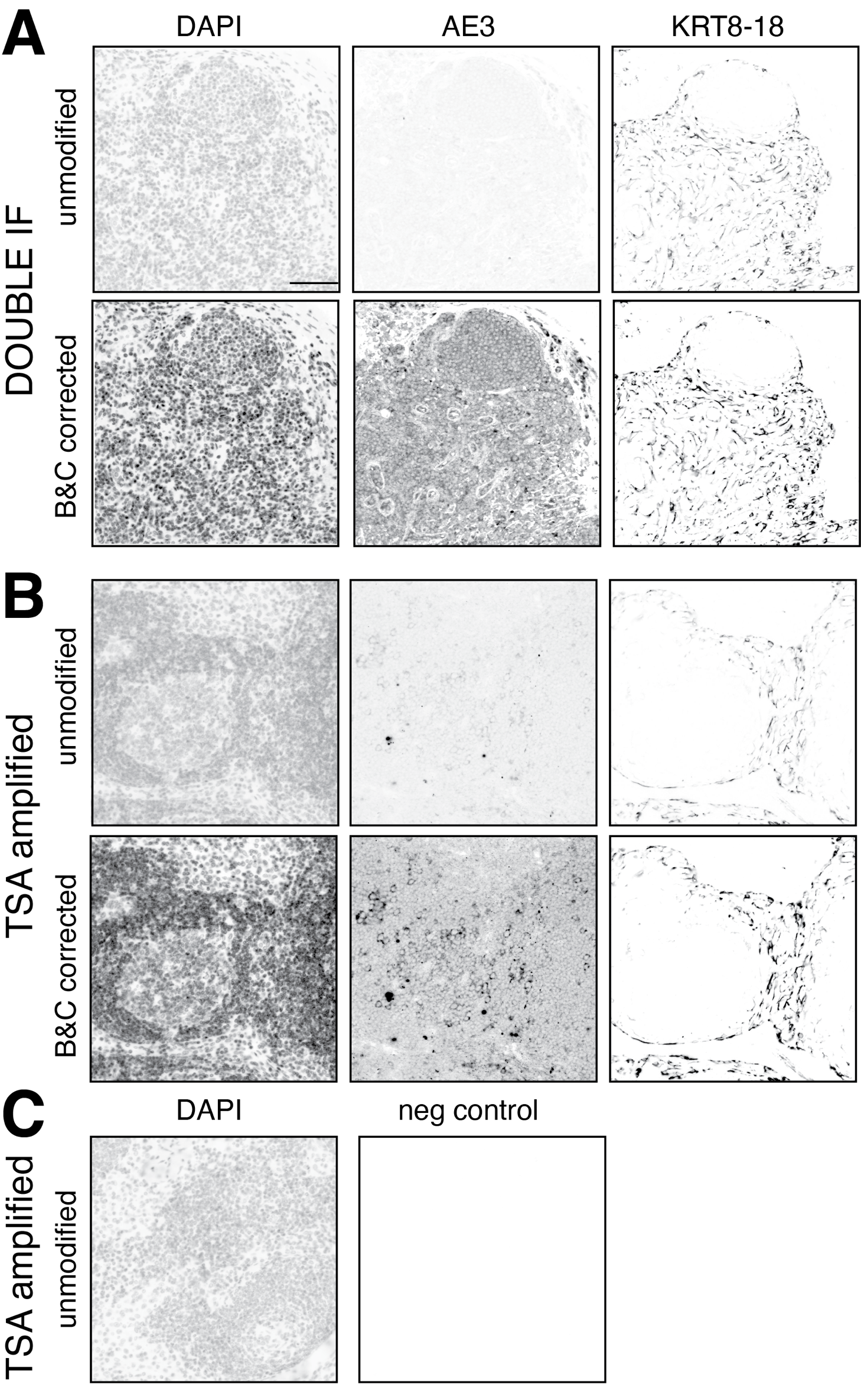


Fig. S8

Comparison of staining efficiency and image manipulation for CK staining.

CK AE3 and LMW KRT pool staining by double indirect IF (**A**) and by TSA (**B**) are compared. For each method the unmodified (top) and the Brightness and Contrast modifications of inverted IF images (bottom) are shown. **C** A negative control after TSA is shown, unmanipulated. For the image manipulation parameters, see Fig. S9.


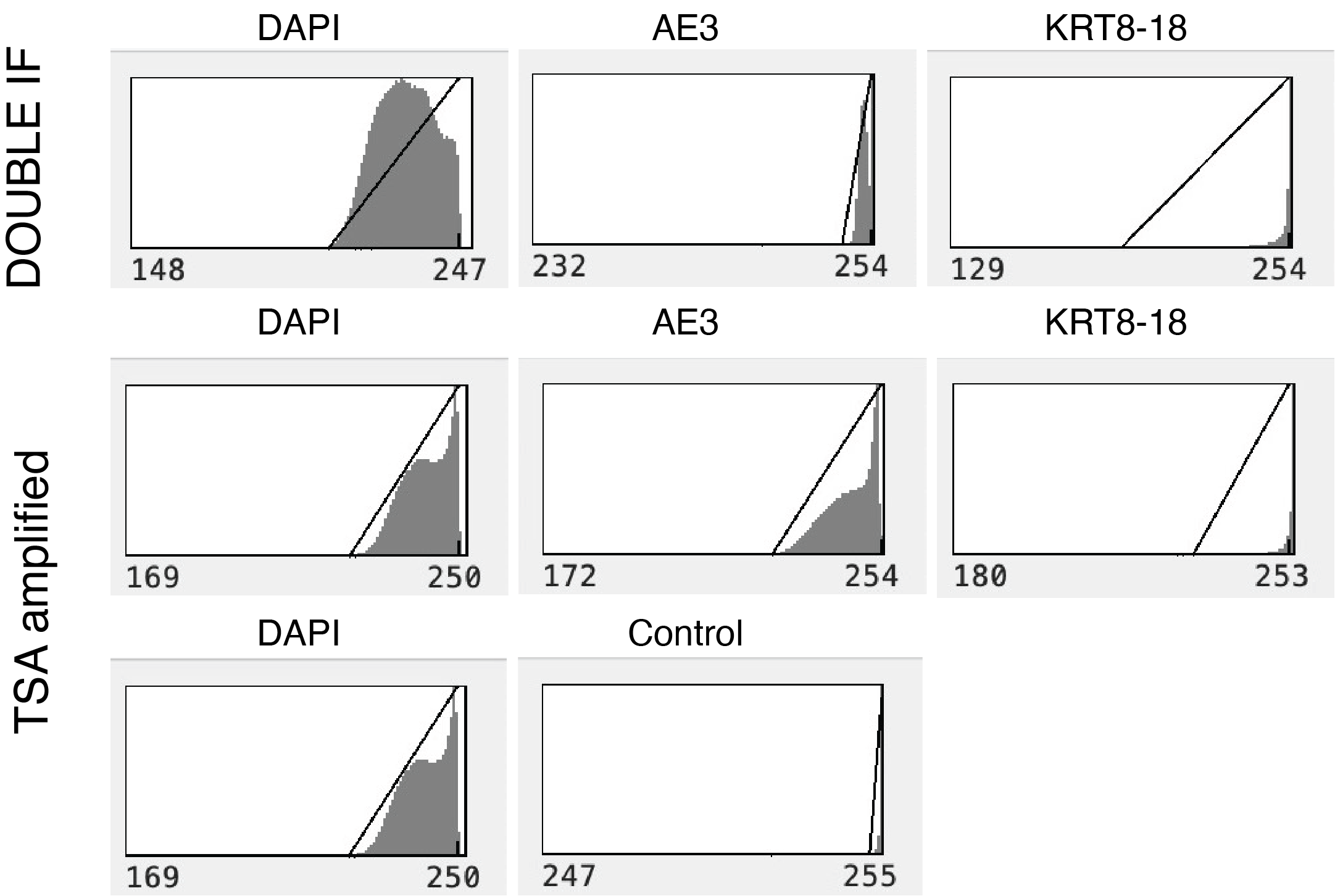


Fig. S9

Brightness & Contrast settings for image S8.

The Brightness & Contrast settings for the corresponding images of Fig. S8 are shown.

SUPPLEMENTARY REFERENCES

1. Li C, Ziesmer S, Lazcano-Villareal O. Use of azide and hydrogen peroxide as an inhibitor for endogenous peroxidase in the immunoperoxidase method. The Journal of Histochemistry and Cytochemistry. 1987;35(12):1457-60.

2. Bolognesi MM, Manzoni M, Scalia CR, Zannella S, Bosisio FM, Faretta M, et al. Multiplex Staining by Sequential Immunostaining and Antibody Removal on Routine Tissue Sections. J Histochem Cytochem. 2017;65(8):431-44.

3. Manzoni M, Bolognesi MM, Antoranz A, Mancari R, Carinelli S, Faretta M, et al. The Adaptive and Innate Immune Cell Landscape of Uterine Leiomyosarcomas. Scientific reports. 2020;10(1):702-10.

4. Liechti T, Weber LM, Ashhurst TM, Stanley N, Prlic M, Van Gassen S, et al. An updated guide for the perplexed: cytometry in the high-dimensional era. Nature Immunology. 2021:1-8.
